# Effects of Polysaccharide Elicitors from Endophytic *Fusarium oxysporum* Fat9 on the Growth, Flavonoid Accumulation and Antioxidant Property of *Fagopyrum tataricum* Sprout Cultures

**DOI:** 10.3390/molecules21121590

**Published:** 2016-11-25

**Authors:** Lingyun Zhong, Bei Niu, Lin Tang, Fang Chen, Gang Zhao, Jianglin Zhao

**Affiliations:** 1 Department of Biological Sciences, College of Life Sciences, Sichuan University, Chengdu 610065, Sichuan, China; zhongly@cdu.edu.cn (L.Z.); tanglinscu11@163.com (L.T.); 2 National R&D Center for Coarse Cereal Processing, Chengdu University, Chengdu 610106, Sichuan, China; niubeicdu@163.com (B.N.); ccpczhaogang@163.com (G.Z.)

**Keywords:** polysaccharide, endophyte *Fusarium oxysporum* Fat9, tartary buckwheat, flavonoid, elicitation, antioxidant activity

## Abstract

The purpose of this study was to evaluate the effects of four different fungal polysaccharides, named water-extracted mycelia polysaccharide (WPS), sodium hydroxide-extracted mycelia polysaccharide (SPS), hydrochloric-extracted mycelia polysaccharide (APS), and exo-polysaccharide (EPS) obtained from the endophytic *Fusarium oxysporum* Fat9 on the sprout growth, flavonoid accumulation, and antioxidant capacity of tartary buckwheat. Without visible changes in the appearance of the sprouts, the exogenous polysaccharide elicitors strongly stimulated sprout growth and flavonoid production, and the stimulation effect was closely related with the polysaccharide (PS) species and its treatment dosage. With application of 200 mg/L of EPS, 200 mg/L of APS, 150 mg/L of WPS, or 100 mg/L of SPS, the total rutin and quercetin yields of buckwheat sprouts were significantly increased to 41.70 mg/(100 sprouts), 41.52 mg/(100 sprouts), 35.88 mg/(100 sprouts), and 32.95 mg/(100 sprouts), respectively. This was about 1.11 to 1.40-fold compared to the control culture of 31.40 mg/(100 sprouts). Moreover, the antioxidant capacity of tartary buckwheat sprouts was also enhanced after treatment with the four PS elicitors. Furthermore, the present study revealed the polysaccharide elicitation that caused the accumulation of functional flavonoid by stimulating the phenylpropanoid pathway. The application of beneficial fungal polysaccharide elicitors may be an effective approach to improve the nutritional and functional characteristics of tartary buckwheat sprouts.

## 1. Introduction

**Figure 1:**
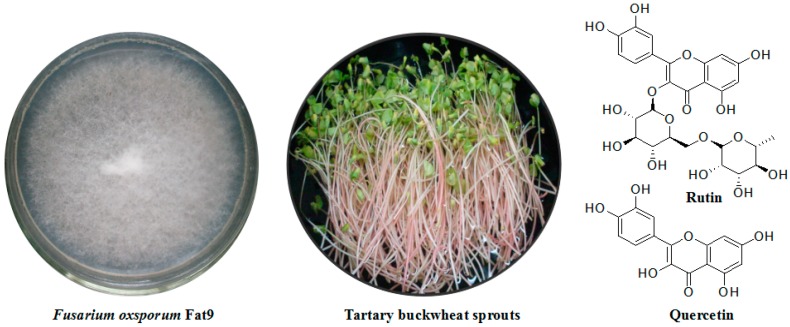
Endophyte *F. oxysporum* Fat9, sprouts and main flavonoids of *F. tataricum*.

Tartary buckwheat (*Fagopyrum tataricum* (L.) Gaertn, Polygonaceae), a pronounced medicinal and edible crop, has been widely planted around the world for food and drink [1,2]. It is rich in protein, amino acids, dietary fiber, vitamins, trace elements, as well as various beneficial phytochemicals. The major bioactive components of tartary buckwheat are flavonoids, phenolics, steroids, fagopyrins, and d-chiro-inositol. These components have demonstrated notable antioxidant, hypocholesterolemic, antidiabetic, antimicrobial and antitumor properties [3,4,5], and this has attracted many researchers’ interest. As a result, greater interest has been paid to the development of buckwheat products recently. More and more novel buckwheat products are introduced every year such as bread, cake, noodles, sauce, sprouts, tea, vinegar, and wine. Among these products, buckwheat sprouts (Figure 1) are becoming very popular in China, Japan, South Korea, and many European countries [6,7,8,9].

Buckwheat sprouts, which have a soft, slightly crispy texture and an attractive fragrance, have been regarded as a nutritional and healthy vegetable in recent years [8,9]. Because sprouting improves the protein quality and fatty acid composition of tartary buckwheat, its sprouts are more nutritious and healthy than their seeds. Furthermore, the biosynthesis of many functional metabolites such as rutin, quercetin, γ-aminobutyric acid and d-chiro-inositol, which have a variety of pharmacological effects, are also notably enhanced [9,10,11].

As the accumulation of many secondary metabolites in plants is usually a defense response of plants to biotic or abiotic stresses, their biosynthesis can be stimulated by various elicitors [12]. During the past few years, the application of non-pathogenic fungal preparations or chemicals as elicitors has become one of the most important and successful strategies for enhancing functional metabolite production in plant tissue cultures. Such elicitors mainly consisted of living or autoclaved fungi mycelia, crude extracts, proteins, peptides, as well as fungal carbohydrates, etc. [12,13,14].

Plant endophytic fungi are a special group of microorganisms that reside within plant tissues intercellularly or intracellularly without causing any apparent symptoms of disease. During the long period of co-evolution, a symbiotic relationship has developed between each endophyte and its host plant [15,16]. The endophytic fungi may produce many functional constituents such as antimicrobial, insecticide, cytotoxic, and growth regulator agents, etc. They may protect the host from pathogen attacks, promote host growth, improve adaption, and help the host to keep healthy [16,17]. Fungal endophytes have been known as an important resource for natural bioactive compounds, and show great potential for applications in the agriculture, food and medicine industries. Research on endophytic fungi has become a hotspot in recent years. Nevertheless, there are few reports about the effects of endophytic fungi as elicitors of growth and bioactive metabolite biosynthesis of their host plants. 

In our previous study, the crude mycelia extract of endophytic fungus *F. oxysporum* Fat9 promoted the growth and flavonoid production of tartary buckwheat sprouts [18]. The aim of the present study was to investigate the effects of four different kinds of fungal polysaccharides (i.e., WPS, SPS, APS and EPS) prepared from endophytic *F. oxysporum* Fat9, on the growth, functional flavonoid accumulation, and antioxidant capacity of tartary buckwheat sprout cultures. In addition, the intracellular phenylalanine ammonia lyase (PAL) activity of buckwheat sprouts induced by fungal polysaccharides was investigated further, and the potential relationship to plant stress response is also discussed.

## 2. Results and Discussion

### 2.1. Effects of WPS, SPS, APS and EPS on the Growth of Tartary Buckwheat Sprout Cultures

The effects of four endophytic fungal polysaccharide elicitors (WPS, SPS, APS and EPS) on seed germination and sprout growth of tartary buckwheat are listed in Table 1, and are closely related with the PS species and elicitation dosage. On the whole, the germination of buckwheat seeds was effectively stimulated by the three polysaccharides WPS (100–200 mg/L), APS (100–300 mg/L), and EPS (50–300 mg/L). The SPS elicitor had no significant positive effect on the seed germination. Among these elicitation treatments, the highest germination rate was 97.33% when the seeds were treated with 150 mg/L of EPS, about 1.18-fold compared to the control culture of 82.67%. The buckwheat sprout length was also efficiently promoted by WPS, APS, and EPS elicitors, and their length was between 13.37 cm and 14.63 cm, about 1.07 to 1.17-fold compared to the control (12.47 cm). However, the SPS elicitor exhibited a slight or negative effect on the sprout length of tartary buckwheat with its higher treatment doses (150–400 mg/L). 

For the sprout biomass, all four of the PS elicitors (WPS, SPS, APS and EPS) could enhance the sprout growth with suitable elicitation dosages. With these effective elicitors, the fresh weight of buckwheat sprout was from 14.25 g/(100 sprouts) to 16.35 g/(100 sprouts), about 1.04- to 1.19-fold in comparison with the control of 13.71 g/(100 sprouts). Correspondingly, the maximum dry weight of buckwheat sprout was 1.01 g/(100 sprouts), when treated with 150 mg/L of EPS. This was about 1.15-fold compared to the control culture of 0.88 g/(100 sprouts).

### 2.2. Effects of WPS, SPS, APS and EPS on Rutin and Quercetin Accumulation of Buckwheat Sprouts

**Figure 2:**
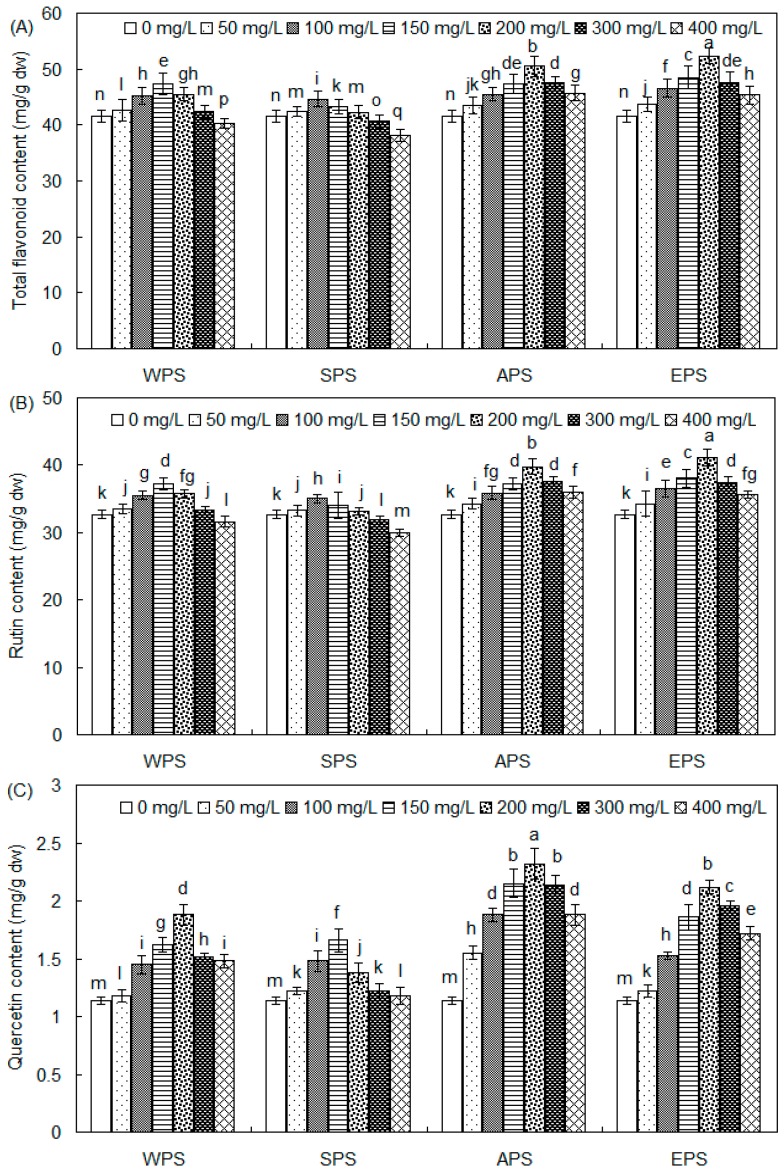

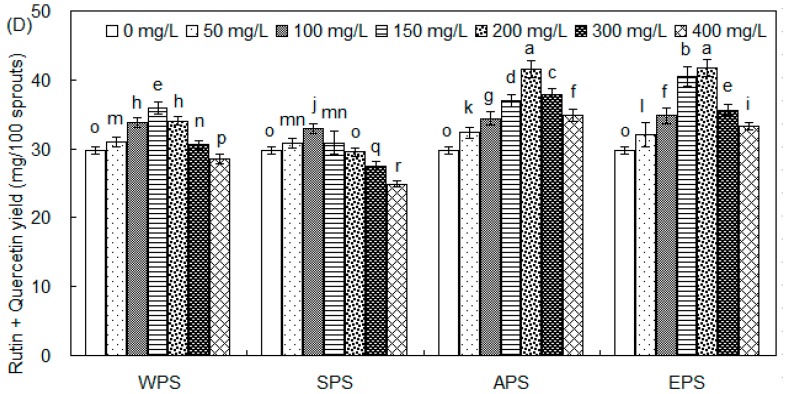
Effects of WPS, SPS, APS and EPS (0, 25, 50, 100, 200 and 400 mg/L) of endophyte *F. oxysporum* Fat9 on the total flavonoid content (**A**), rutin content (**B**), quercetin content (**C**), and total rutin and quercetin yield (**D**) of tartary buckwheat sprout cultures (*n* = 3). Different letters (i.e., a–r) in each column indicated significant differences among the treatment at *p* = 0.05 level.

The effects of four polysaccharide elicitors on total flavonoid, rutin, and quercetin production are displayed in Figure 2. As shown in Figure 2A, the total flavonoid accumulation of tartary buckwheat sprouts was efficiently stimulated by all four PS elicitors. The highest flavonoid content of buckwheat sprout cultures was 52.36 mg/g dw with 200 mg/L of EPS, about 1.26-fold in comparison with the control culture of 41.58 mg/g dw. After treatment with 200 mg/L of APS, the total flavonoid content of buckwheat sprouts was increased to 50.48 mg/g dw. With 150 mg/L of WPS, the total flavonoid content of buckwheat sprouts was as much as 47.36 mg/g dw. 

Rutin biosynthesis was stimulated by the WPS, APS, and EPS elicitors (Figure 2B). The maximum rutin content of tartary buckwheat sprouts was 41.11 mg/g dw when treated with 200 mg/L of EPS, about 1.25-fold compared to the control of 32.65 mg/g dw. After treatment with 200 mg/L of APS, the sprouts rutin content was increased to 39.78 mg/g dw. In addition, the rutin content of buckwheat sprouts was as much as 37.18 mg/g dw with 150 mg/L of WPS. Quercetin production was significantly promoted by all four polysaccharides. With these effective elicitors, the quercetin content of buckwheat sprout cultures was elevated from 1.48 mg/g dw to 2.32 mg/g dw, about 1.30 to 2.07-fold in comparison with the control culture of 1.14 mg/g dw (Figure 2C). Correspondingly, the highest rutin and quercetin yield of tartary buckwheat sprouts was 41.70 mg/(100 sprouts), 41.52 mg/(100 sprouts), 35.88 mg/(100 sprouts), and 32.95 mg/(100 sprouts), when the sprouts were treated with 200 mg/L of EPS, 200 mg/L of APS, 150 mg/L of WPS, and 100 mg/L of SPS, respectively, about 1.11 to 1.40-fold compared with the control culture of 29.75 mg/(100 sprouts) (Figure 2D). 

### 2.3. Antioxidant Activity Evaluation of Tartary Buckwheat Sprouts after Treatment with WPS, SPS, APS and EPS

**Figure 3:**
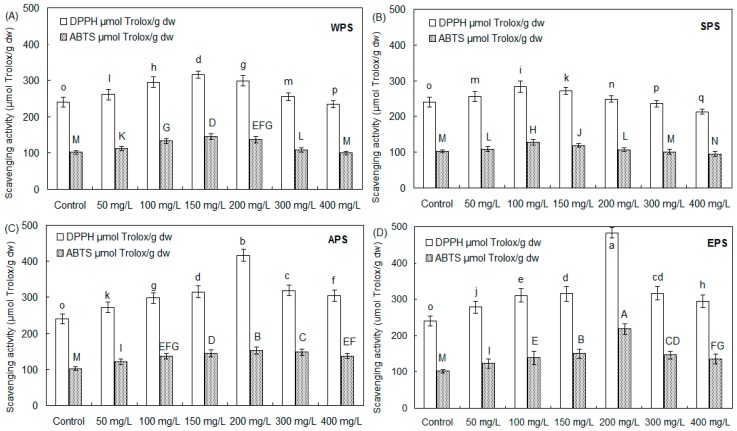
DPPH and ABTS radical scavenging activity of tartary buckwheat sprouts after treatment with WPS (**A**), SPS (**B**), APS (**C**)and EPS (**D**) elicitors of endophyte *F. oxysporum* Fat9 in comparison with the control culture (*n* = 3). Different letters (i.e., a–q, A–N) in each column indicated significant differences among the treatment at *p* = 0.05 level.

The 1,1-diphenyl-2-picrylhydrazyl radical (DPPH^●^), and 2,2’-azinobis-3-ethylbenzthiazoline- 6-sulfonate radical cation (ABTS^●+^) scavenging capacities of tartary buckwheat sprout cultures treated with WPS, SPS, APS and EPS are shown in Figure 3. In general, the four polysaccharide elicitors of endophyte *F. oxysporum* Fat9 enhanced the antioxidant activity of buckwheat sprouts, and the stimulatory effect was concentration-dependent. Of them, the buckwheat sprouts treated with 200 mg/L of EPS exhibited the strongest DPPH scavenging capacity of 483.52 μmol Trolox/g dw, about 2.01-fold compared with the control culture of 240.36 μmol Trolox/g dw (Figure 3D). Subsequently, the scavenging activity of buckwheat sprouts elicited with 200 mg/L of APS, 150 mg/L of WPS, and 100 mg/L of SPS was 416.38 μmol Trolox/g dw (Figure 3C), 315.68 μmol Trolox/g dw (Figure 3A), and 283.53 μmol Trolox/g dw (Figure 3B), respectively. The ABTS^●+^ scavenging activity of tartary buckwheat sprout cultures had a similar pattern as the DPPH radical scavenging capacity. The buckwheat sprouts treated with 200 mg/L of EPS had the highest scavenging activity of 218.85 μmol Trolox/g dw, about 2.14-fold in comparison with the control of 102.36 μmol Trolox/g dw (Figure 3D). Then, for the buckwheat sprouts applied with 200 mg/L of APS, 150 mg/L of WPS, and 100 mg/L of SPS, its scavenging capacity was determined as 152.95 μmol Trolox/g dw (Figure 3C), 145.18 μmol Trolox/g dw (Figure 3A) and 128.36 μmol Trolox/g dw (Figure 3B), respectively.

**Figure 4:**
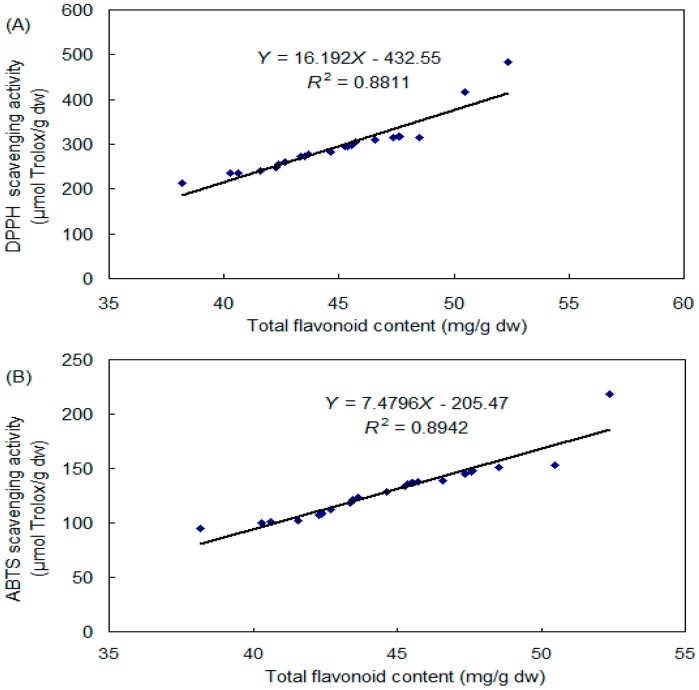
Correlations between flavonoid concentration and DPPH radical scavenging capacity (**A**), and ABTS radical cation scavenging capacity (**B**).

The correlation analysis between total flavonoid concentration and DPPH radical scavenging activity, and ABTS^●+^ scavenging capacity are displayed in Figure 4. The positive correlation between the two variables is indicated by the correlation coefficients (*r*). When the absolute value of *r* is close to 1, there is strong correlation. The *r* value of DPPH, and ABTS^●+^ scavenging ability with total flavonoid is calculated as 0.9387 and 0.9456, respectively, suggesting that rutin and quercetin are the major anti-oxidative constituents, and there are strong correlations between flavonoid and DPPH/ABTS^●+^ scavenging capacity. These results are in accordance with previous reports [7,19,20].

### 2.4. Kinetics of Sprout Growth, Flavonoid Accumulation and PAL Activity after Treatment with WPS, SPS, APS and EPS

**Figure 5:**
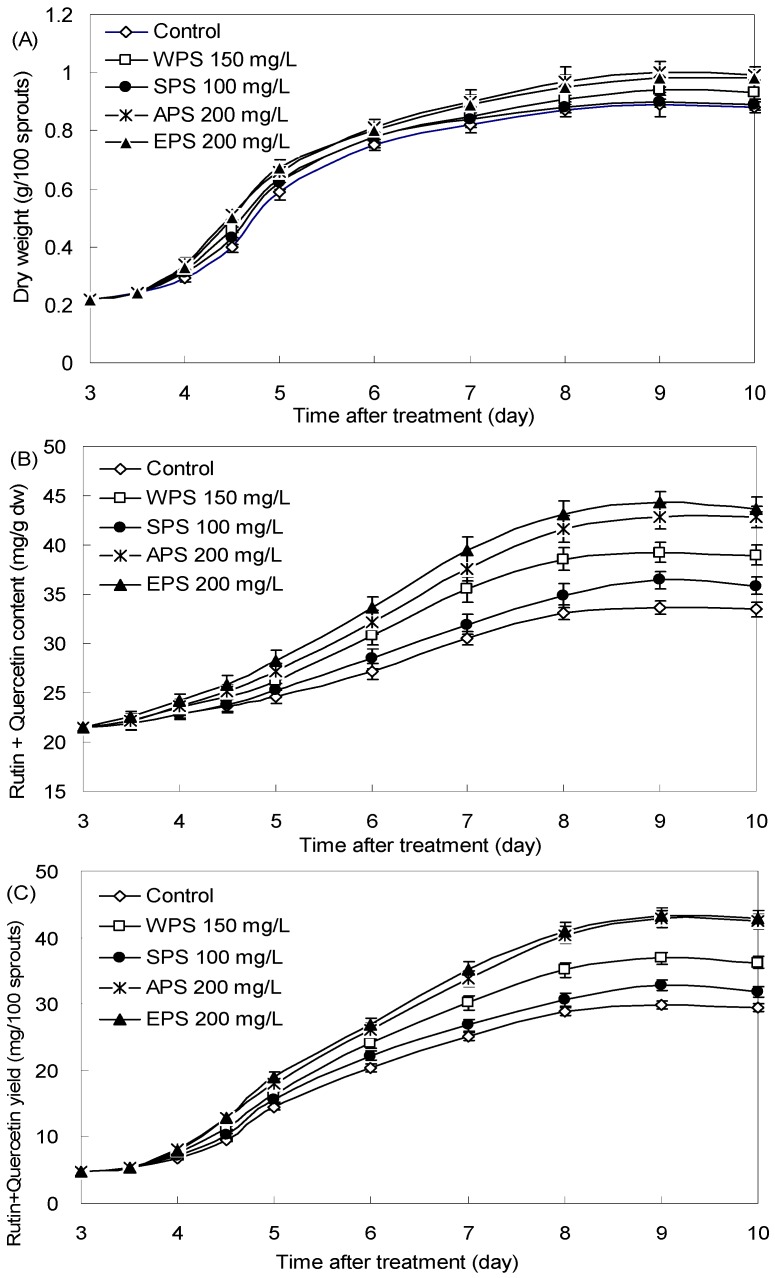
Kinetic studies of sprout growth (**A**), rutin and quercetin accumulation (**B**), and rutin and quercetin production (**C**) of tartary buckwheat after treatment with 150 mg/L of WPS, 100 mg/L of SPS, 200 mg/L of APS and 200 mg/L of EPS of endophyte *F. oxysporum* Fat9 in comparison with the control culture (*n* = 3).

According to the results mentioned above, the four elicitors WPS (150 mg/L), and SPS (100 mg/L), APS (200 mg/L), and EPS (200 mg/L) strongly stimulated flavonoid production in tartary buckwheat sprouts. Consequently, kinetic studies of tartary buckwheat sprout growth and rutin and quercetin biosynthesis stimulated by these effective elicitors were further investigated, which are displayed in Figure 5. Generally, the promoting effects of four optimal PS elicitors on the sprout growth could be clearly observed after elicitation treatments for two days (Figure 5A). The highest sprout biomass was 1.02 g/(100 sprouts) for 200 mg/L of APS, about 1.15-fold in comparison with the control culture of 0.89/(100 sprouts). The maximum buckwheat sprout dry weight was 0.98 g/(100 sprouts) for 200 mg/L of EPS, 0.94 g/(100 sprouts) for 150 mg/L of WPS, and 0.91 g/(100 sprouts) for 100 mg/L of SPS obtained on day 9. 

As shown in Figure 5B, the stimulation effects of WPS, SPS, APS and EPS on rutin and quercetin accumulation could be noticed significantly three days after elicitation treatments, and then they steadily increased to the end of the culture period. The highest rutin and quercetin content of tartary buckwheat sprout cultures was 44.26 mg/g dw obtained on day 9 for 200 mg/L of EPS, which was about 1.32-fold compared to the control of 33.62 mg/g dw. This was 44.86 mg/g dw for 200 mg/L of APS, 39.22 mg/g dw for 150 mg/L of WPS, and 36.43 mg/g dw for 100 mg/L of SPS, respectively (Figure 5B). Correspondingly, the maximum rutin and quercetin yield of buckwheat sprout was 43.37 mg/(100 sprouts) achieved on day 9 with 200 mg/L of EPS, about 1.45-fold in comparison with the control culture of 29.92 mg/(100 sprouts), 42.86 mg/(100 sprouts) for 200 mg/L of APS, 36.87 mg/(100 sprouts) for 150 mg/L of WPS, and 32.79 mg/(100 sprouts) for 100 mg/L of SPS, respectively (Figure 5C).

**Figure 6:**
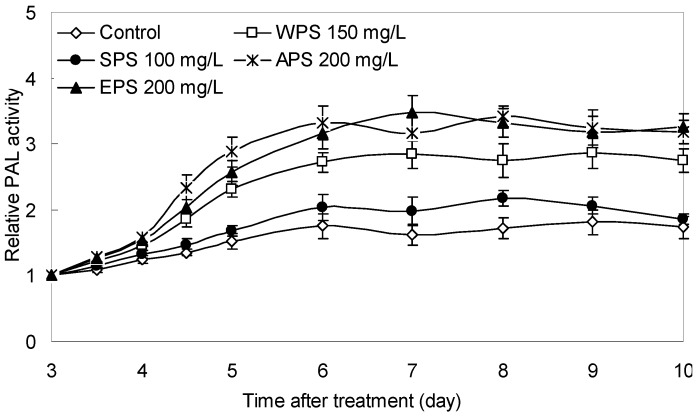
Time courses of PAL activity of tartary buckwheat sprouts after treatment with 150 mg/L of WPS, 100 mg/L of SPS, 200 mg/L of APS and 200 mg/L of EPS of endophyte *F. oxysporum* Fat9 compared to the control culture (*n* = 3).

As shown in Figure 6, the PAL activity of tartary buckwheat sprouts was notably induced by the three optimal fungal PS (200 mg/L of EPS, 200 mg/L of APS and 150 mg/L of WPS) elicitation treatments, from 1.14 to 2.15-fold of the control level. Although the SPS (100 mg/L) elicitor exhibited a slight stimulation effect on the PAL activity of the buckwheat sprouts, it was only about 1.05- to 1.27-fold of the control culture over the treatment period. It is well known that PAL is a critical enzyme at the entrance step in the phenylpropanoid pathway in plants, and that elicitors increased its activity suggests an enhanced secondary metabolism in plant cell and tissue cultures [9,21,22]. Based on present study results, it could be speculated that the phenylpropanoid pathway is closely related to flavonoid biosynthesis in tartary buckwheat sprout cultures. This is consistent with previous reports [9,18,23]. Moreover, it also suggests that the exogenous fungal polysaccharides (EPS, APS and WPS) obtained from endophyte *F. oxysporum* Fat9 may be recognized as effective biotic elicitors and absorbed by receptors on the surface of the sprouts or transformed to a stress signal stimulating functional flavonoid biosynthesis in buckwheat sprouts. In any case, these valuable findings provide further evidence for fungal polysaccharides elicitor activity stimulating responses and secondary metabolism in tartary buckwheat sprout cultures.

## 3. Experimental

### 3.1. Cultivation of Endophytic Fungus Fusarium oxysporum Fat9

The endophytic fungus *Fusarium oxysporum* Fat9 (GenBank accession number KC218453, and CGMCC 10102) was isolated from the healthy plant of tartary buckwheat as reported previously [18]. The living culture has been maintained on potato dextrose agar (PDA) slants at 4 °C, and in 40% glycerol at −70 °C at the coarse cereal processing center (CCPC) of Chengdu University, City, Country. After the fungal mycelia were grown on PDA medium in Petri dishes at 25 °C for 4–6 days, two to three agar plugs with mycelia were transplanted and grown in a 500 mL Erlenmeyer flask containing 200 mL liquid potato dextrose (PD) medium. All shake flasks were maintained on a rotary shaker (Ruichang, Sichuan, China) at 150 rpm and 25 °C for seven days. A total of 25 L fermentation broth was prepared and centrifuged at 3000 rpm for 20 min. Then, the supernatant and mycelia were collected, respectively.

### 3.2. Preparation of EPS, WPS, SPS and APS

**Figure 7:**
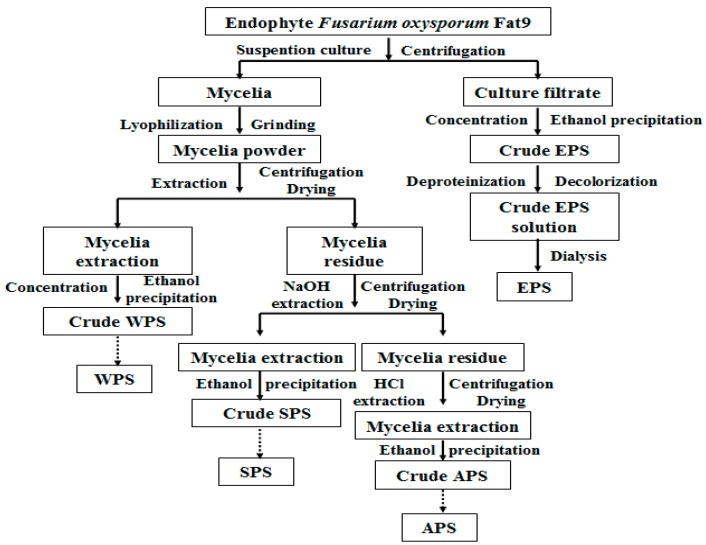
The preparation scheme of EPS, WPS, SPS, and APS of endophyte *F. oxysporum* Fat9.

The preparation scheme of four different fungal polysaccharides of endophytic fungus *F. oxysporum* Fat9 was shown in Figure 7. The exo-polysaccharide (EPS) of endophyte *F. oxysporum* Fat9 was prepared from the supernatant mentioned above. In brief, the supernatant was concentrated under vacuum at 50–55 °C by a rotary evaporator (Yarong, Shanghai, China) to a suitable volume, mixed with three volumes of 95% ethanol, and allowed to precipitate for 48 h at 4 °C in a refrigerator. After that, the solution was centrifuged at 8000 rpm for 15 min, and the precipitate was collected as crude EPS, which was further subjected to deproteinization with Sevag reagent, decolorization with 3% H_2_O_2_, and removal of small molecule impurities by dialysis. Polysaccharide mixture with molecular weight greater than 8000–14,000 Da remained in the dialysis tube. After lyophilization, the purified EPS (6.82 g) was kept in a desiccator (Shuniu, Sichuan, China). The carbohydrate concentration of EPS was measured by the anthrone test using glucose as a reference [22], and its content was determined as 91.3%.

The collected mycelia of *F. oxysporum* Fat9 was first washed three times with deionized water, and then lyophilized. The lyophilized mycelia was powdered and subjected to heat circumfluence extraction at 50 °C by 95% ethanol–petroleum ether at 1:1 (*v*/*v*) to remove the lipid, mono-saccharide and disaccharide. The ratio of mycelia powder (g) to refluxing solvent (mL) was 1:6 (*w*/*v*). The defatted mycelia powder was obtained by centrifugation and drying at 40–45 °C to a constant weight. Then, the mycelia powder was immersed in distilled water, and extracted at 85 °C for 90 min with the ratio of the material (g) to water (mL) as 1:20 (*w*/*v*). Afterwards, centrifugation was performed at 8000 rpm for 15 min to separate the supernatant and the residue. The supernatant was concentrated to a certain volume, and mixed with three volumes of 95% ethanol, and then kept in a refrigerator at 4 °C for 48 h. The following procedure for polysaccharide purification was the same as the treatments of EPS. The obtained polysaccharide (7.63 g) was named as WPS. The residue not containing WPS was further extracted with 1 M sodium hydroxide (NaOH) solution at root temperature for 24 h. The remaining steps were the same as the treatments of WPS. The prepared polysaccharide (6.85 g) was designated as SPS. The residue not containing WPS and SPS was further extracted with 1 M HCl solution at room temperature for 24 h. The remaining steps were the same as the treatments of WPS mentioned above. The obtained polysaccharide (7.46 g) was named as acid-extracted mycelial polysaccharide (APS). The carbohydrate content of WPS, SPS and APS was determined as 87.5%, 81.3% and 90.6%, respectively.

### 3.3. Application of EPS, WPS, SPS and APS to the Buckwheat Sprout Cultures

The stock polysaccharide solutions were prepared by dissolving each polysaccharide in distilled water, and sterilized by filtering through a microfilter (0.45 μm). The effects of four PS elicitors (WPS, SPS, APS and EPS) on the buckwheat sprout growth, flavonoid accumulation, and antioxidant activity were investigated following elicitation treatments. The healthy buckwheat seeds (cultivar chuanqiao-02) were pre-surface-sterilized for 5 min in 2.5% sodium hypochlorite solution followed by a quick deionized-H_2_O rinse three times. Then, they were immersed in each polysaccharide solution in the following seven concentrations (0, 50, 100, 150, 200, 300 and 400 mg/L) for 12 h, respectively, and transplanted into commercial germination boxes (120 mm × 120 mm × 50 mm). The buckwheat sprouts were cultivated in illumination incubators at 25 ± 1 °C and 70% relative humidity, and harvested on day 9 or 10 for measuring their germination rate, sprout length, biomass, flavonoid content, and antioxidant activity. Moreover, the kinetics of optimal PS-treated buckwheat sprout growth and flavonoid biosynthesis was also studied, and the four PS elicitors were applied to the buckwheat sprout cultures on day 3, when the sprout formed and its length was nearly 1–2 cm. All treatments were carried out in triplicate.

### 3.4. Measurement of Germination Rate, Sprout Biomass and Flavonoid Content

At the end of the culture period, the germination rate of buckwheat seeds was counted. The percentage (%) of seed germination was determined as (*G*/*t*) × 100, where *G* is an average number of three replicates of germinated seeds, and *t* is an average value of three replicates of the total seeds in each test. For measuring the sprout biomass, they were harvested and rinsed thoroughly with distilled water, and blotted dry by paper towels to obtain the fresh weight (fw), and then dried at 40–45 °C in an oven (Taihua, Jiangsu, China) to attain the constant dry weight (dw). 

To measure flavonoid (rutin and quercetin) content of tartary buckwheat sprouts, the dried spouts were ground into powder with a pestle and mortar, and the extraction was carried out by mixing buckwheat sprout powder (0.1 g) with a methanol-water (25 mL, 70%, *v*/*v*) solution in a conical flask under ultrasonic processing for 30 min. After filtration, the filtrates were transferred into a 25 mL volumetric flask and the volume adjusted to 25 mL with 70% methanol. The rutin and quercetin content were analyzed by HPLC, according to our previous method [18]. In addition, the total flavonoid content in buckwheat sprouts was determined using the aluminium chloride colorimetric method described by Chang [24]. Briefly, the appropriate dilution of extractions (1 mL) was mixed with 2 mL of 10% aluminium chloride, 3 mL of 1 M potassium acetate, and 5 mL of 70% methanol. After incubation at room temperature for 30 min, the absorbance of the reaction mixture was measured at 420 nm against a methanol blank on a UV-3200S Spectrophotometer (Mapada, Shanghai, China). The total flavonoid content was calculated using a standard calibration of rutin solution and expressed as micrograms of rutin equivalent per gram of sample. Standard calibration was made from 6.25, 12.5, 25.0, 50.0 and 100 μg/mL.

### 3.5. DPPH and ABTS Radical-Scavenging Activity Assays

The DPPH radical-scavenging capacities of tartary buckwheat sprouts were evaluated by the reduction of the reaction color between DPPH solution and sample extracts as described previously [7,25]. Briefly, 1 mL of the extraction or control was mixed with 4.0 mL of 0.124 mg/mL DPPH radical solution. The mixture was shaken vigorously and left to stand at room temperature for 30 min in the dark. Then, the mixture was measured spectrophotometrically at 517 nm against a methanol blank. A standard of Trolox was run using several concentrations ranging from 17.08 μmol/L to 273.25 μmol/L. The scavenging activity was calculated using the following equation:

DPPH radical-scavenging ability (%) = [(A_0_ − A_S_)/A_0_] × 100, (1)

where A_0_ = absorbance of DPPH radical + methanol, A_S_ = absorbance of DPPH radical + flavonoid extract. A standard curve was then prepared by plotting the percentage (%) of free radical scavenging activity of Trolox versus its concentration. The final result was expressed as μmol Trolox equivalent antioxidant capacity in 1 g of sample (μmol Trolox eq./g DW). 

The ABTS^●+^ scavenging activity assay of buckwheat sprouts was evaluated according to the method of Peng [7] with slight modification. ABTS^●+^ was dissolved and adjusted to 5.0 mmol/L with phosphate buffer (pH 7.4), then oxidized with manganese dioxide, and left to stand under ambient temperature over night. The final reaction mixture contained 3.0 mL of ABTS^●+^ with an absorbance of 0.7 adjusted by phosphate buffer (pH 7.4) at 734 nm, and 200 μL of the appropriate dilutions of extracts or 200 μL of distilled water for the control. The absorbance at 734 nm was measured after a reaction time of 1 min. The final result was expressed as μmol Trolox eq./g DW that was calculated using a standard curve prepared with Trolox. Data was recorded as mean ± SD for three replicates.

### 3.6. Measurement of PAL Activity

The phenylalanine ammonia lyase (PAL) was extracted from the fresh tartary buckwheat sprouts with borate buffer (pH 8.8). Afterwards, the sprouts were ground in the buffer (0.2 g/mL) for 2 min with a sterilized pestle and mortar on ice, and the homogenate was then centrifuged at 8000 rpm and 4 °C for 20 min to obtain a solid-free extract for quantification. Subsequently, the PAL activity was measured by the colorimetric method as described previously by Zhao with slight modifications, which is based on the conversion of *L*-phenylalanine to cinnamic acid [14].

### 3.7. Statistical Analysis

All elicitation treatments were carried out three times, and the results were expressed by their mean values and standard deviations (SD). The experimental data were submitted to analysis of their variance to determine significant differences by PROC ANOVA of SAS version 9.2 (SAS Institute Inc., Cary, NC, USA). The term significant difference was based on *p* ≤ 0.05.

## 4. Conclusions

In conclusion, this study demonstrated the beneficial effects of polysaccharide (PS) elicitors obtained from endophytic *Fusarium oxysporum* Fat9 on the growth, flavonoid accumulation and antioxidant property of tartary buckwheat sprouts. Without visible changes in sprout appearance, the four exogenous fungal PS (EPS, APS, WPS, and SPS) strongly stimulated the flavonoid biosynthesis of buckwheat sprouts, and the stimulation effect was concentration-dependant. With application of 200 mg/L of EPS, 200 mg/L of APS, 150 mg/L of WPS, or 100 mg/L of SPS on the sprout cultures, the total rutin and quercetin content was effectively increased to 52.36 mg/g dw, 50.48 mg/g dw, 47.36 mg/g dw, and 44.65 mg/g dw, which was about 1.07 to 1.26-fold in comparison with the control culture of 41.58 mg/g dw. Moreover, the antioxidant capacity of tartary buckwheat sprouts was also notably enhanced after treatment with these four efficient PS elicitors. Furthermore, the present study also disclosed that the accumulation of flavonoid in tartary buckwheat sprout cultures resulted mainly from the stimulation of the phenylpropanoid pathway by PS elicitation treatments. These findings indicate that polysaccharides from endophyte *F. oxysporum* Fat9 are potent elicitors and growth-promoting constituents for tartary buckwheat sprout cultures. Nevertheless, the chemical composition of these polysaccharides (especially for EPS and APS), the structure-function relationship, the physiological responses and molecular mechanisms of the sprouting induced by polysaccharides, the process optimization for polysaccharide production, as well as the effects of PS on the growth and flavonoid biosynthesis of tartary buckwheat in the field need to be further clarified and studied.

## Figures and Tables

**Table 1:** Effects of four fungal polysaccharide elicitors WPS, SPS, APS and EPS on the sprout growth of tartary buckwheat.

Treatment	Polysaccharide Concentration (mg/L)	Germination Rate (%)	Sprout Length (cm)	Fresh Weight (g/100 Sprouts)	Dry Weight (g/100 Sprouts)
Control	0	82.67 ± 1.53 ^g^^,h,i^	12.47 ± 0.15 ^j,k^	13.71 ± 0.04 ^h^	0.88 ± 0.01 ^f,g,h,i^
WPS	50	84.00 ± 1.00 ^f^^,g,h^	12.57 ± 0.21 ^i,j^	14.09 ± 0.12 ^g^	0.89 ± 0.01 ^e,f,g,h,i^
	100	87.33 ± 1.15 ^d,e^	12.87 ± 0.15 ^f,g,h^	14.28 ± 0.07 ^f,g^	0.91 ± 0.03 ^d,e,f^
	150	90.33 ± 1.53 ^c^	13.37 ± 0.15 ^e^	14.55 ± 0.27 ^e^	0.92 ± 0.01 ^c,d,e,f^
	200	85.00 ± 1.00 ^e,f,g^	12.77 ± 0.15 ^g,h,i^	14.23 ± 0.11 ^f,g^	0.90 ± 0.01 ^e,f,g,h^
	300	81.33 ± 1.15 ^i^	12.40 ± 0.10 ^j,k,l^	13.33 ± 0.09 ^i^	0.88 ± 0.01 ^f,g,h,i^
	400	75.33 ± 1.15 ^k^	12.23 ± 0.12 ^k,l^	13.14 ± 0.03 ^i^	0.86 ± 0.01 ^h,i,j^
SPS	50	83.67 ± 0.58 ^f,g,h,i^	12.57 ± 0.06 ^i,j^	14.07 ± 0.04 ^g^	0.89 ± 0.01 ^e,f,g,h,i^
	100	86.00 ± 1.00 ^d,e,f^	12.80 ± 0.10 ^g,h,i^	14.25 ± 0.12 ^f,g^	0.90 ± 0.01 ^e,f,g,h^
	150	82.00 ± 1.00 ^h,i^	12.37 ± 0.12 ^j,k,l^	13.22 ± 0.04 ^i^	0.87 ± 0.02 ^g,h,i,j^
	200	78.00 ± 2.00 ^j^	12.13 ± 0.06 ^l^	12.85 ± 0.09 ^j^	0.85 ± 0.01 ^i,j^
	300	74.33 ± 1.15 ^k^	11.47 ± 0.15 ^m^	12.35 ± 0.11 ^k^	0.83 ± 0.01 ^j,k^
	400	69.67 ± 1.53 ^l^	10.67 ± 0.12 ^n^	11.55 ± 0.02 ^l^	0.80 ± 0.01 ^k^
APS	50	86.00 ± 1.00 ^d,e,f^	12.63 ± 0.12 ^h,i,j^	14.24 ± 0.13 ^f,g^	0.90 ± 0.01 ^e,f,g,h^
	100	87.67 ± 2.08 ^d^	13.13 ± 0.21 ^e,f^	14.27 ± 0.12 ^f,g^	0.91 ± 0.01 ^e,f^
	150	91.67 ± 1.53 ^b,c^	13.77 ± 0.15 ^c,d^	15.38 ± 0.13 ^d^	0.94 ± 0.02 ^c,d,e^
	200	96.33 ± 1.53 ^a^	14.53 ± 0.12 ^a^	15.95 ± 0.24 ^b^	0.99 ± 0.04 ^a,b^
	300	90.33 ± 2.52 ^c^	13.97 ± 0.15 ^b,c^	15.60 ± 0.21 ^c^	0.96 ± 0.02 ^b,c,d^
	400	85.33 ± 0.58 ^d,e,f^	13.63 ± 0.15 ^d^	14.69 ± 0.08 ^e^	0.92 ± 0.01 ^d,e,f^
EPS	50	92.33 ± 1.15 ^b,c^	13.00 ± 0.26 ^f,g^	14.26 ± 0.12 ^f,g^	0.90 ± 0.01 ^e,f,g,h^
	100	93.67 ± 1.15 ^b^	13.30 ± 0.26 ^e^	14.34 ± 0.08 ^f^	0.91 ± 0.01 ^d,e,f^
	150	97.33 ± 1.15 ^a^	14.63 ± 0.15 ^a^	16.35 ± 0.09 ^a^	1.01 ± 0.08 ^a^
	200	92.00 ± 1.00 ^b,c^	14.13 ± 0.21 ^b^	15.65 ± 0.11 ^c^	0.96 ± 0.01 ^b,c^
	300	87.00 ± 1.00 ^d,e^	12.97 ± 0.21 ^f,g^	14.33 ± 0.09 ^f^	0.91 ± 0.02 ^e,f,g^
	400	84.33 ± 0.58 ^f,g,h^	12.57 ± 0.06 ^i,j^	14.09 ± 0.08 ^g^	0.89 ± 0.02 ^f,g,h,i^

Values represent mean ± standard deviation (*n* = 3). Different letters (i.e., a–n) in each column indicated significant differences among the treatment at *p* = 0.05 level.

## References

[1] Fabjan N., Rode J., Košir I.J., Wang Z.H., Zhang Z., Kreft I. (2003). Tartary buckwheat (*Fagopyrum tataricum* Gaertn.) as a source of dietary rutin and quercitrin. J. Agric. Food Chem..

[2] Zhang Z.L., Zhou M.L., Tang Y., Li F.L., Tang Y.X., Shao J.R., Xu W.T., Wu Y.M. (2012). Bioactive compounds in functional buckwheat food. Food Res. Int..

[3] Inglett G.E., Chen D.J., Berhow M., Lee S. (2011). Antioxidant activity of commercial buckwheat flours and their free and bound phenolic compositions. Food Chem..

[4] Ren Q., Li Y.F., Wu C.S., Wang C.H., Jin Y., Zhang J.L. (2014). Metabolism of secondary metabolites isolated from tartary buckwheat and its extract. Food Chem..

[5] Zhu F. (2016). Chemical composition and health effects of Tartary buckwheat. Food Chem..

[6] Gimenez-Bastida J.A., Pisku1a M., Zielinski H. (2015). Recent advances in development of gluten-free buckwheat products. Trends Food Sci. Tech..

[7] Peng L.X., Zou L., Wang J.B., Zhao J.L., Xiang D.B., Zhao G. (2015). Flavonoids, antioxidant activity and aroma compounds analysis from different kinds of tartary buckwheat tea. Indian J. Pharm. Sci..

[8] Kim S.L., Kim S.K., Park C.H. (2004). Introduction and nutritional evaluation of buckwheat sprouts as a new vegetable. Food Res. Int..

[9] Kim H.J., Park K.J., Lim J.H. (2011). Metabolomic analysis of phenolic compounds in buckwheat (*Fagopyrum esculentum* M.) sprouts treated with methyl jasmonate. J. Agric. Food Chem..

[10] Wang L., Li X.D., Niu M., Wang R., Chen Z.X. (2013). Effect of additives on flavonoids, d-chiro-inositol and trypsin inhibitor during the germination of tartary buckwheat seeds. J. Cereal Sci..

[11] Ghimeray A.K., Sharma P., Phoutaxay P., Salitxay T., Woo S.H., Park S.U., Park C.H. (2014). Far infrared irradiation alters total polyphenol, total flavonoid, antioxidant property and quercetin production in tartary buckwheat sprout powder. J. Cereal Sci..

[12] Ramirez-Estrada K., Vidal-Limon H., Hidalgo D., Moyano E., Golenioswki M., Cusidó R.M., Palazon J. (2016). Elicitation, an effective strategy for the biotechnological production of bioactive high-added value compounds in plant cell factories. Molecules.

[13] Li J.X., Liu S.J., Wang J., Li J., Liu D.H., Li J.L., Gao W.Y. (2016). Fungal elicitors enhance ginsenosides biosynthesis, expression of functional genes as well as signal molecules accumulation in adventitious roots of *Panax ginseng* C. A. Mey. J. Biotechnol..

[14] Zhao J.L., Zou L., Zhong L., Peng L.X., Ying P.L., Tan M.L., Zhao G. (2015). Effects of polysaccharide elicitors from endophytic *Bionectria pityrodes* Fat6 on the growth and flavonoid production in tartary buckwheat sprout cultures. Cereal Res. Commun..

[15] Venugopalan A., Srivastava S. (2015). Endophytes as in vitro production platforms of high value plant secondary metabolites. Biotechnol. Adv..

[16] Zhao J., Shan T., Mou Y., Zhou L. (2011). Plant-derived bioactive compounds produced by endophytic fungi. Mini-Rev. Med. Chem..

[17] Rodriguez R.J., White J.F., Amold A.E., Redman R.S. (2009). Fungal endophytes: Diversity and functional roles. New Phytol..

[18] Zhao J., Zhong L.Y., Zou L., Zhang C., Peng L., Xiao W., Zhao G. (2014). Efficient promotion of the sprout growth and rutin production of tartary buckwheat by associated fungal endophytes. Cereal Res. Commun..

[19] Yao Y.P., Tian C.R., Cao W. (2008). Anti-oxidative constituents of ethanol extract from buckwheat seeds by HPLC-Elecro-Spray MS. Agric Sci. Chin..

[20] Lu X., Wang L., Wer H., Yang Z.Q., Wang W. (2006). Structure-activity relationship of flavonoids in antioxidant activity. Food Sci..

[21] Zhao J., Zhou L., Wu J. (2010). Effects of biotic and abiotic elicitors on cell growth and tanshinone accumulation in *Salvia miltiorrhiza* cell cultures. Appl. Microbiol. Biotechnol..

[22] Zhao G., Zhao J.L., Peng L.X., Zou L., Wang J.B., Zhong L.Y., Xiang D.B. (2012). Effects of yeast polysaccharide on growth and flavonoid accumulation in *Fagopyrum tataricum* sprout cultures. Molecules.

[23] Liu J.F., Li X.Y., Meng R. (2006). Preliminary studies on the factors for promoting flavonoids production during the germination process of tatary buckwheat. Sci. Technol. Food Ind..

[24] Chang C.C., Yang M.H., Wen H.M., Chern J.C. (2002). Estimation of total flavonoid content in propolis by two complementary colorimetric methods. J. Food Drug Anal..

[25] Guo X.D., Ma Y.J., Parry J., Gao J.M., Yu L.L., Wang M. (2011). Phenolics content and antioxidant activity of tartary buckwheat from different locations. Molecules.

